# Mechanistic modeling of gastrointestinal motility with integrated dissolution for simulating drug absorption

**DOI:** 10.5599/admet.829

**Published:** 2020-06-09

**Authors:** Kevin C. Johnson

**Affiliations:** Intellipharm, LLC, 47 Magazine Street, Cambridge, MA 02139

**Keywords:** PBPK modeling, gastric emptying, drug particle size, multiple moving plug model

## Abstract

A new computational method – the multiple moving plug (MMP) model – is described to simulate the effect of gastrointestinal motility and dissolution on the pharmacokinetic profile of any given drug. The method is physiologically more consistent with the experimental evidence that fluid exists in discrete plugs in the gastrointestinal tract, and therefore is more realistic than modeling the gastrointestinal tract as a series of compartments with first-order transfer. The number of plugs used in simulations, their gastric emptying times and volumes, and their residence times in the small intestine can be matched with experimental data on motility. In sample simulations, drug absorption from a series of fluid plugs emptied from the stomach at evenly spaced time intervals showed lower C_max_ and higher T_max_ than an equivalent dose emptied immediately as a single plug. To the extent that new techniques can establish typical ranges for the volumes of fluid emptied from the stomach and their respective timing, the MMP model may be able to predict the effect of gastric emptying on the variability seen in pharmacokinetic profiles. This could lead to an expanded safe space for the regulatory acceptance of formulations based on dissolution data.

## Introduction

Several reports have expressed the view that gastrointestinal (GI) transit is more realistically modeled as discrete plugs of fluid emptying from the stomach and moving through the small intestine rather than as a first-order process between static compartments [1-3]. Experimental evidence comes from magnetic resonance imaging showing isolated pockets of fluid in the GI tract [4-6]. Two of the reports [1, 2] describe stochastic models for gastric emptying, but these models omit the effects of drug dissolution, absorption, and metabolism on plasma drug concentrations. By contrast, the multiple moving plug (MMP) model [3] is a natural extension of a well-established dissolution model [7-9], the default model in GastroPlus [10], and can simulate the effects of both the gastric emptying of plugs and the dissolution within the plugs on plasma drug concentrations. The MMP model is capable of utilizing either experimental data or stochastic simulations of the volumes and timing of plugs emptying from the stomach. The computational method of the MMP model is described here in more rigorous mathematical detail than previously discussed [3]. Sample simulations show the effects of gastric emptying on the pharmacokinetics of a hypothetical drug.

## Computational method

**Figure 1.**
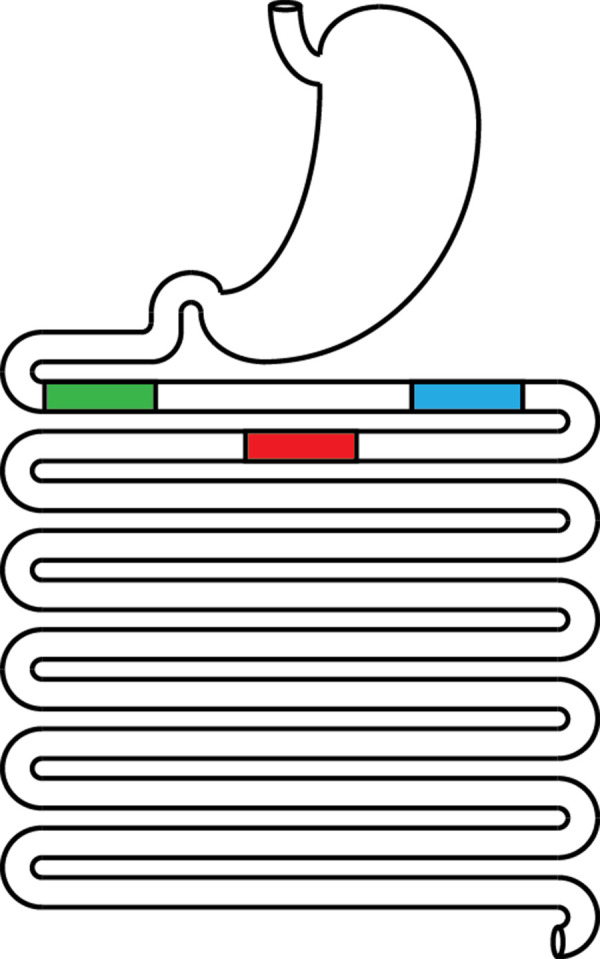
Drawing of the GI tract illustrating multiple moving plugs of fluid. In the simulations for Figures 2-4, the first plug (j=1) to empty from the stomach at time zero is shown in red, the second (j=2) at 15 minutes is shown in blue, and the third (j=3) at 30 minutes is shown in green.

A system of coupled differential equations describing drug dissolution, absorption, and metabolism was solved numerically using the fourth-order Runge-Kutta method. Development of this system has been described previously as well as the derivation of the Noyes-Whitney equation assuming spherical drug particle geometry [3, 7, 8]. The equations used are summarized in Table 1 including the definitions of variables and parameters. New is the addition of the index *j* to create and track discrete plugs that move independently through the GI tract as illustrated in Figure 1. It should be noted that Figure 1 was drawn to show a reasonably realistic length-to-diameter ratio for the small intestine and was untangled to facilitate this perspective [11]. In reality, the small intestine must be much more convoluted to fit into the small space of the abdominal cavity. Independence is achieved by making plug-specific initial conditions and time-/position-dependent changes to variables associated with each plug. Increasing the number of plugs allows more refined simulation of localized GI events and the resulting variability in drug plasma concentrations. A specific example of the Microsoft Visual Basic code used for the simulations is available at https://intellipharm.com/wp-content/uploads/2020/04/mmp.txt. Reference 3 provides a general explanation of the coding techniques applied to the MMP model.

A step size of 0.001 minute was used for the Runge-Kutta method. The iterative nature of this method allows any parameter to be changed at a frequency equal to the step size. Equations 1-4 describe how drug mass changes in various locations, but other parameters can be changed to match the physical characteristics of the location. For example, it has already been described how *h*_ij_ changes with a change in drug particle radius as particles dissolve [3, 7, 8]. Because the number of parameter changes that could be simulated is unlimited, only *k*_a_j__ was altered to explore the potential effect of gastric emptying on the drug plasma concentration profile. Table 2 lists the other parameters used in the simulations that were held constant. They are similar to the values reported for nifedipine [12].

In keeping with the generally accepted view that the main site of drug absorption is the small intestine and not the stomach [13], *k*_a_j__ was assumed to be zero while plug j was in the stomach. The number of plugs exiting the stomach could be any number *n*_p_, and the volume of each plug *V*_j_ could be any fraction of the total volume containing any fraction of the total dose. For simplicity, each *V*_j_ was equal to 240 mL divided by *n*_p_ (240 mL/3 = 80 mL).

To simulate gastric emptying, *k*_a_j__ was transitioned from its value of zero in the stomach to its value of 0.07 min^-1^ in the duodenum. The transition time was assumed to be 1 minute. Table 3 shows the equation and parameters used to simulate a smooth sigmoidal transition for the value of *k*_a_j__, but any appropriate function could be used to accomplish the transition. A simulation using immediate changes for *k*_a_j__ instead of smooth transitions was essentially the same as the original simulation. However, smooth transitions are recommended as abrupt changes can lead to instabilities in the numerical method.

The function described by Equation 8 in Table 3 is bounded by two pair of points: (*t_k_*_a_j_st_, *k*_a_j_st_) and (*t_k_*_a_j_si_, *k*_a_j_si_). The number of transitions can be increased by using Equation 8 and common points as the start and end points of transitions. The time course of transitions taking place within a given plug can be made to be completely independent of that in any other plug. If the initial conditions and transitions that define each plug are exactly the same, then the simulation would be equivalent to a single plug of the equivalent total dose and volume.

Modeled as a series of discrete fluid plugs exiting the stomach, each containing drug particles in the process of dissolving, the effect of gastric emptying and drug particle size on drug pharmacokinetics was simulated for three evenly-spaced plugs. The total dose and fluid volume were held constant, but the drug particle size and dose in individual plugs were changed between simulations as shown in Table 4. In each simulation, the first plug began to transition from the stomach to the small intestine immediately, the second beginning at 15 minutes, and the third beginning at 30 minutes.

## Results and Discussion

**Figure 2.**
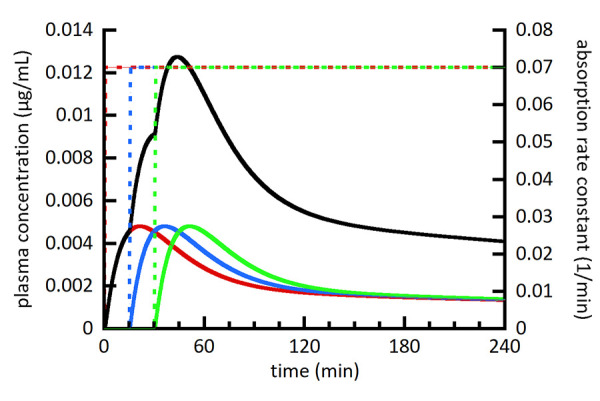
Simulated plasma drug concentration (left axis) from 3 individual plugs (red, blue, and green solid lines) as well as the summation of all plugs (black solid line). Absorption from individual plugs begins at the onset of the absorption rate constant transitioning from 0 to 0.07 min^-1^ shown as dashed lines and scaled using the right axis. Table 4 lists figure specific simulation parameters.

**Figure 3.**
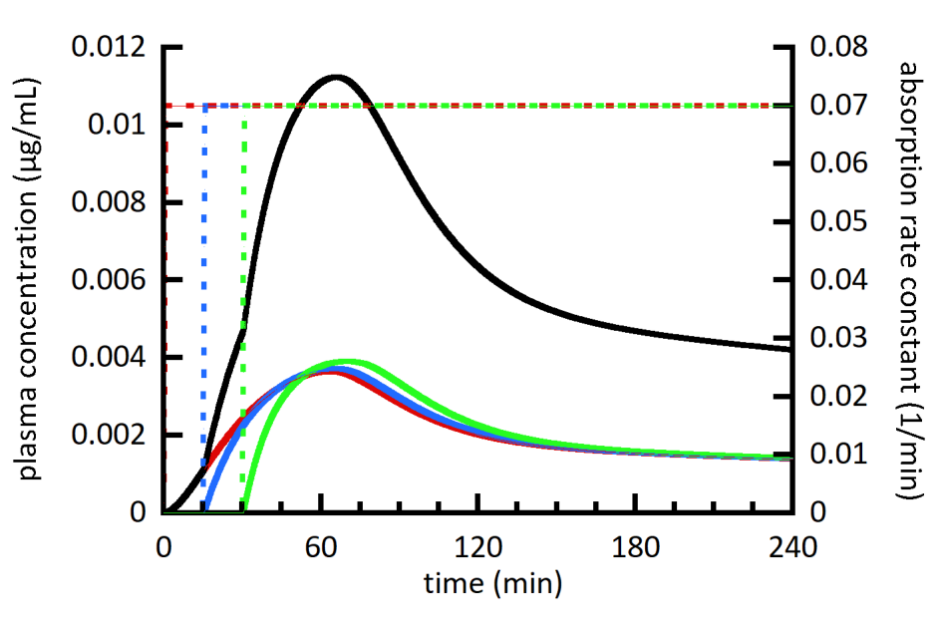
Simulated plasma drug concentration (left axis) from 3 individual plugs (red, blue, and green solid lines) as well as the summation of all plugs (black solid line). Absorption from individual plugs begins at the onset of the absorption rate constant transitioning from 0 to 0.07 min^-1^ shown as dashed lines and scaled using the right axis. Table 4 lists figure specific simulation parameters. Figure 3 uses a larger drug particle size compared to Figure 2.

**Figure 4.**
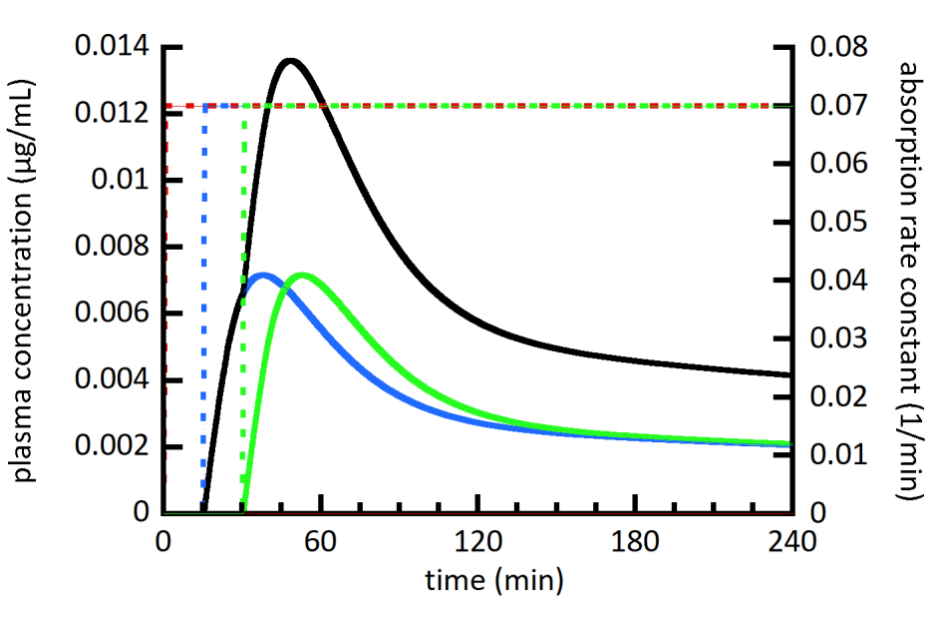
Simulated plasma drug concentration (left axis) from 3 individual plugs (red, blue, and green solid lines) as well as the summation of all plugs (black solid line). Absorption from individual plugs begins at the onset of the absorption rate constant transitioning from 0 to 0.07 min^-1^ shown as dashed lines and scaled using the right axis. Table 4 lists figure specific simulation parameters. Figure 4 removes the amount of drug in the first plug and splits it evenly between the second and third plugs compared to Figure 2.

Figures 2-4 show a series of simulations using the multiple moving plug model. The main goal was to provide a visual and conceptual understanding of the model and its capabilities within a limited number of simulations. With this in mind, the number of plugs (3), their individual volumes (80 mL), and the timing of their gastric emptying (0, 15, and 30 min) were held constant. The spacing of plug emptying times was intentionally exaggerated to allow visual distinction between the plugs. To make more accurate simulations, the number, volumes, and emptying times of plugs should be selected to agree with emerging experimental measurements. Figures 2-4 show the individual plasma compartment drug concentrations arising from drug absorption from individual plugs as well as the total drug concentration from all plugs. The solid lines represent plasma concentrations using the left axis and dashed lines represent absorption rate constants using the right axis. For the absorption rate constant versus time profiles, the sigmoidal shape of the transition representing gastric emptying is difficult to discern, but it should be noted that it is not an abrupt change.

In Figure 2, the concentration profiles from individual plugs appear to have the same shape. This is because dissolution is essentially complete for all plugs when they are simulated to empty from the stomach. In comparison, the individual profiles in Figure 3 are clearly different due to the slower dissolution rate from larger drug particles. In Figure 3, the first plug (red) has significantly less drug dissolved than the last plug (green) when they empty from the stomach. This results in a slower rate of drug absorption from the first plug compared to the last plug and illustrates the ability of the MMP model to model plugs independently.

Figure 4 illustrates the ability of the MMP model to simulate inhomogeneity in the stomach by assigning different initial conditions to various plugs. The situation depicted in Figure 4 might arise if a dosage form does not disintegrate immediately after ingestion with water. In this case, the first plug of fluid to empty from the stomach does not contain any drug. Instead, the 0.8 mg of drug in the first plug in Figure 2 was divided evenly between the second and third plugs. As expected, there was a delay in the onset of drug absorption in Figure 4 relative to Figure 2. The simulation in Figure 4 also resulted in a higher *C*_max_ for the total drug concentration compared to Figure 2.

**Figure 5.**
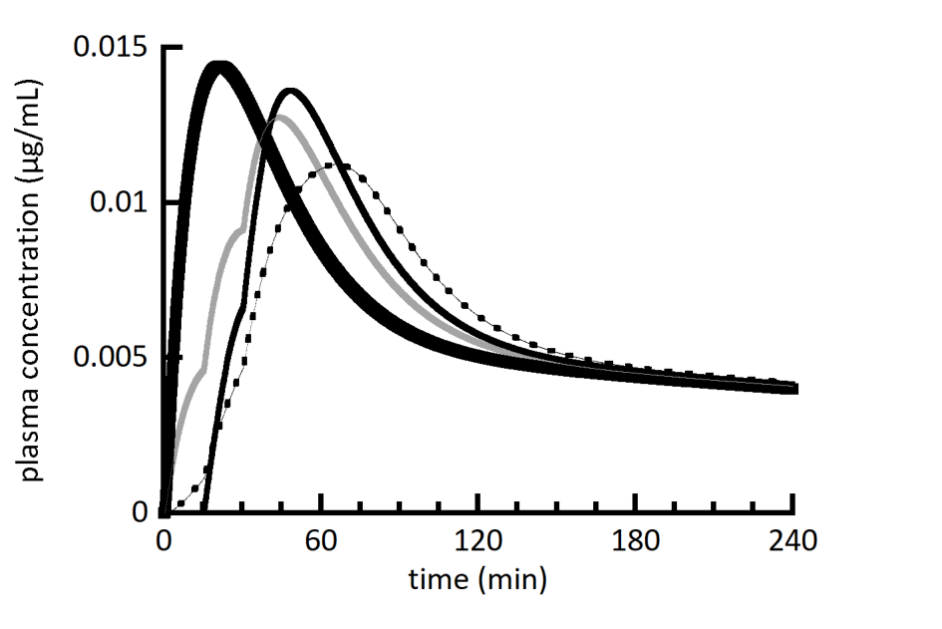
Simulated plasma drug concentration (summation only) from Figure 2 (gray line), Figure 3 (dashed line), and Figure 4 (solid thinner line) compared to a one-plug simulation for reference (solid heavier line) that emptied from the stomach using the absorption rate constant transition for the first plug in Figures 2-4.

Figure 5 compares the summation plasma concentration profiles in Figures 2-4 as well as a new simulation for absorption of the same total dose from a single plug to serve as a reference point. In general, simulating gastric emptying as a sequential movement of a series of independent plugs of fluid from the stomach to the intestine resulted in a reduced *C*_max_ and increased *T*_max_ compared to a single plug.

The MMP model invokes the need for real time data on the number, timing, and volume of fluid plugs emptying from the stomach and moving through the intestine. Schiller *et al*. [6] reported a mean small intestinal fluid volume of 105 mL contained in a mean number of separated fluid pockets of 4. However, this assessment was a one-time observation made 1 hour after the last dose in a sequence of non-disintegrating marker capsules was given in the fasting state. Mudie *et al*. [4] tracked the number, volume, and approximate location of fluid packets at fixed time points. After dosing 240 mL of water in the fasted state, the number of small intestinal water pockets increased from about 8 initially to about 16 before decreasing gradually over the 2-hour time period studied. The mean volume of the pockets was in the 3-7 mL range. Mudie *et al*. [4] acknowledge that it is possible that a significant portion of the dose could be confined to just a few pockets. This suggests that the focus should be on the fluid that is likely to contain drug at the time of dosing and on its movement thereafter.

**Figure 6.**
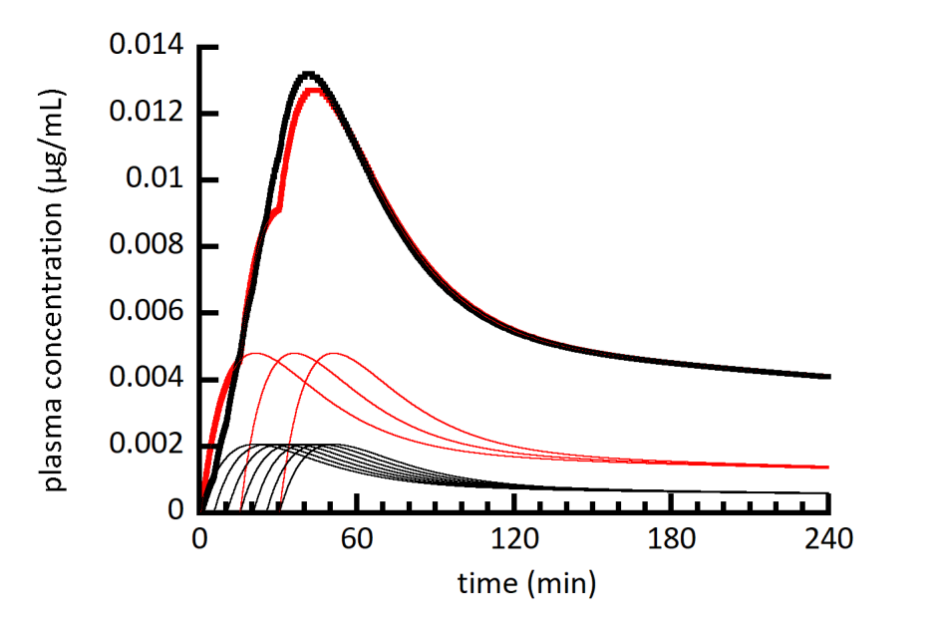
Simulated plasma drug concentration from Figure 2 (red) compared to a new simulation (black) except that the new simulation used 7 plugs instead of 3, and the total dose was divided evenly between 7 plugs instead of 3. The heavier lines represent summations from the lighter individual plug lines.

It may also be possible to group fluid that empties from the stomach over a certain time range to reduce the number of fluid plugs while retaining the characteristics of the MMP model. The advantage in doing so is to reduce the computational time required to run a simulation. The same strategy has been applied successfully for simulating the dissolution of polydisperse drug powders by grouping drug particles within a certain size range into a single size group that is representative of the range. Figure 6 compares the simulation in Figure 2 with a simulation where the only differences are an increase in the number of plugs from 3 to 7 and a decrease in the dose in each plug from one third of the total dose to one seventh. The total plasma concentration profiles from the two simulations were not substantially different, indicating that there is potential to reduce the number of plugs without changing the simulation significantly. The number of plugs used in the MMP should be no more than the number observed experimentally and potentially only a fraction thereof.

The summation technique of the MMP model is similar to the superposition principle, except that the summation occurs at each step of the numerical method, as opposed to summing the contributions after all plugs have been simulated. This treatment allows the MMP model to handle non-linear pharmacokinetics. Different metabolic rates can be tied to the continuously updated drug mass in different locations: the intestine for gut wall metabolism, the portal vein for first-pass hepatic metabolism, or the plasma compartment for renal clearance. The pharmacokinetic model described in Table 1 would have to be expanded to allow for the simulation of portal vein drug concentrations.

The advantage of the MMP model is that it provides a more realistic computational approach to simulating gastric emptying and GI motility compared to the advanced compartmental absorption and transit (ACAT) model. Historically, the ACAT model has assumed that both solid and dissolved drug move from compartment to compartment in a first-order process without any concomitant movement of compartmental fluid, which remains static in terms of both position and volume [14]. These assumptions are inconsistent with physical observations and cannot be replicated experimentally. Indeed, the development of dissolution testing apparatus involving more than one dissolution vessels to mimic the stomach and segments of the small intestine rely on the concomitant movement of water and solid and dissolved drug [15]. Ehrlein and Schemann [16] have created a website with links to videos using fluoroscopy that provide visual confirmation of the true nature of GI motility.

The other advantage of the MMP model is that all processes are modeled from the perspective of isolated plugs of moving fluid, making experimental validation of the model components possible. For example, it has already been mentioned that the dissolution component of the MMP model has been established [7, 8] and independently verified [9] for a constant volume of fluid. However, *in vivo*, the volume of the dissolution fluid will change; increasing due to secretion of fluids or decreasing due to water absorption. Testing of the dissolution model to simulate secretion can be accomplished by adding fluid to the dissolution vessel at a known rate. On the other hand, removing fluid to simulate water absorption is more difficult. Evaporation is probably not fast enough to mimic the rate of water absorption from the GI tract, but could be used to test the dissolution model over a narrow range. There is no reason to believe that the principles of the mechanistic dissolution model should fail in extrapolating to higher rates of water removal. Moreover, there is a need to simulate the effect of water absorption on drug dissolution and absorption as the reduction in water volume in the GI tract after dosing has been clearly shown by Mudie *et al*. [4] In either case, water secretion or absorption, the volume change in a dissolution vessel can be measured and corrected at each step of the numerical simulation. The dissolution fluid could also be titrated with physiological buffers, dynamically adjusting the pH-dependent solubility and volume change simultaneously.

The MMP model is completely flexible; any number of plugs containing dissolving drug can empty from the stomach, each having a volume and emptying time to match experimental measurements. The critical physiological parameters are the volume of the plugs, the timing of their emptying, and their residence time in various segments of the intestinal tract. Regional differences in permeability and intestinal wall metabolism can be adjusted to reflect the current position of individual plugs. Although the simulations shown in Figures 2-4 were run with equally spaced plugs of the same volume to demonstrate the model, using real gastric emptying data will enable more accurate simulations.

The MMP model assumes that suspended and dissolved drug are homogeneously mixed within the plug, and that only dissolved drug can leave the plug through permeation of the intestinal membrane. There is no mass transfer between plugs, but plugs could be made to converge or diverge by manipulating the time-dependent parameters. It would also be possible to break up a large volume of fluid into several smaller plugs in order to simulate a stepwise concentration gradient axially from the leading to the trailing end of the plug series.

It has long been recognized that gastric emptying can affect the rate of drug absorption [17, 18]. This rate, characterized by *C*_max_ and *T*_max_, is used to establish bioequivalence when considering a formulation change or when comparing a generic dosage form to an innovator’s product. Simulations show that *C*_max_ and *T*_max_ are sensitive to gastric emptying as modeled by the MMP model. If the bioequivalence criteria are too tight, a dosage form could fail bioequivalence testing due to physiological factors beyond the control of the formulation. To address this problem, Dickinson *et al*. [19] have discussed the concept of a safe space to allow for the effect of physiological factors like gastric emptying on the bioequivalence criteria. Physiologically based pharmacokinetic (PBPK) modeling holds promise for establishing this safe space and for providing confidence in making predictions. The hope is that a simple dissolution test could serve to establish bioequivalence in conjunction with PBPK modeling. The theoretical advantage of the MMP model lies in its mechanistically-based mathematical integration of dissolution, gastric emptying, and intestinal motility in a way that is closer to how these processes occur in the GI tract.

## Conclusions

In conclusion, the MMP model provides a more mechanistically realistic computational tool to study the effects of dissolution, gastric emptying, and intestinal motility on the drug plasma concentration profile. The ability to establish the impact of dissolution and GI motility on bioequivalence criteria may expand safe space for regulatory approval of pharmaceutical products based on limited dissolution data and mechanistically-based simulations.

## Figures and Tables

**Table 1.** Differential equations and symbol definitions for the multiple moving plug model.

quantity being tracked		equation
solid drug mass (mg) in the GI tract in plug j from particle size fraction i		(1) 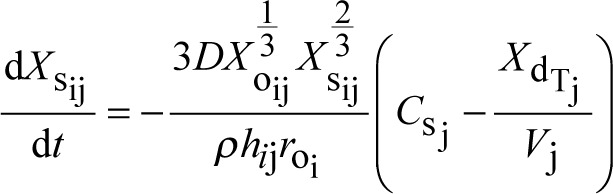
dissolved drug mass (mg) in the GI tract in plug j from particle size fraction i		(2) 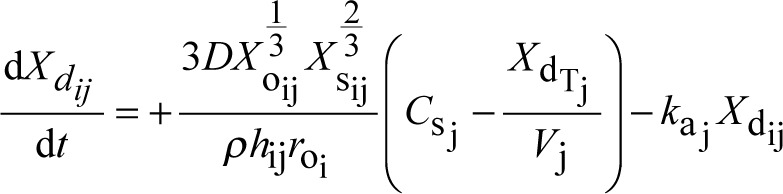
drug mass (mg) in the plasma compartment originating from particle size fraction i in plug j		(3) 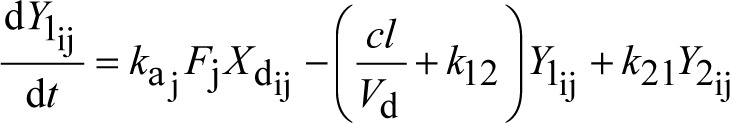
drug mass (mg) in the tissue compartment originating from particle size fraction i in plug j		(4) 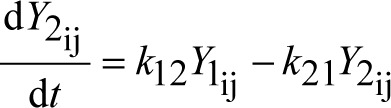
total mass of dissolved drug (mg) in plug j from all particle size fractions		(5) 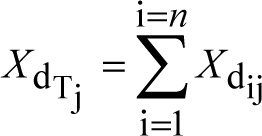
total drug mass (mg) in the plasma compartment from all plugs and all particle size fractions		(6) 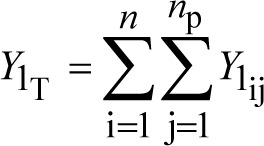
drug concentration (mg/mL) in the plasma compartment		(7) 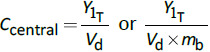
**symbol**	**definition**	
*cl*	clearance (mL/min or mL/min/mg)	
*C*_s_j__	time-dependent drug solubility (mg/mL) for drug in plug j	
*D*	drug diffusion coefficient (cm^2^/min)	
*F*_j_	bioavailability factor for presystemic metabolism in plug j	
*h*_ij_	time-dependent diffusion layer thickness (cm) for particles in plug j from particle size fraction i	
*k*_a_j__	time-dependent absorption rate constant for plug j	
*k*_12_	rate constant for transfer of drug from the plasma to the tissue compartment	
*k*_21_	rate constant for transfer of drug from the tissue to the plasma compartment	
*m*_b_	body mass when *V*_d_ is given in units of volume per unit of body mass	
*n*	number of drug particle size fractions	
*n*_p_	number of plugs	
ρ	drug density (mg/cm^3^)	
*r*__O__i__	initial particle radius (cm) for particles in particle size fraction i	
*t*	time (min)	
*V*_d_	volume of distribution (mL or mL/mg)	
*V*_j_	time-dependent volume (mL) of plug j	
*X*_o_ij__	initial solid drug mass (mg) in the GI tract in plug j from particle size fraction i	

**Table 2.** Parameters used for simulations.

parameter	value
drug solubility (mg/mL)	0.01
total dissolution volume (mL)	240
drug density (g/cm^3^)	1.3
diffusion coefficient (cm^2^/min)	0.0003
body mass (kg)	70
bioavailability factor	0.5
clearance (mL/min/kg)	4
volume of distribution (mL/kg)	600
*k*_12_ (min^-1^)	0.03
*k*_21_ (min^-1^)	0.01

**Table 3.** Equation and symbol definitions used to simulate a smooth transition for the absorption rate constant.

quantity being tracked		equation
Time dependent absorption rate constant for plug j		(8) 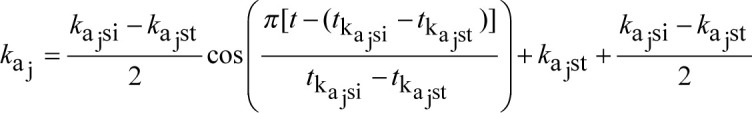
**symbol**	**definition**	
*k*_a_j_si_	absorption rate constant for plug j in the small intestine	
*k*_a_j_st_	absorption rate constant for plug j in the stomach	
*t_k_*_a_j_si_	time at which *k*_a_j__ completes its transition to *k*_a_j_si_ from *k*_a_j_st_	
*t_k_*_a_j_st_	time at which *k*_a_j__ begins its transition from *k*_a_j_st_ to *k*_a_j_si_	

**Table 4.** Plug specific parameters used in Figure 2-4 simulations.

Figure	plug #	gastric emptying onset time (min)	dose in plug (mg)	drug particle size (μm)
2	1	0 (red)	0.8	1
	2	15 (blue)	0.8	1
	3	30 (green)	0.8	1
3	1	0 (red)	0.8	10
	2	15 (blue)	0.8	10
	3	30 (green)	0.8	10
4	1	0 (red)	0	1
	2	15 (blue)	1.2	1
	3	30 (green)	1.2	1
